# Bacterial Community of the Rice Floodwater Using Cultivation-Independent Approaches

**DOI:** 10.1155/2018/6280484

**Published:** 2018-01-30

**Authors:** Michele Pittol, Erin Scully, Daniel Miller, Lisa Durso, Lidia Mariana Fiuza, Victor Hugo Valiati

**Affiliations:** ^1^Programa de Pós-Graduação em Biologia, Universidade do Vale do Rio dos Sinos (UNISINOS), 950 Unisinos Avenue, São Leopoldo, RS, Brazil; ^2^United States Department of Agriculture (USDA), Agricultural Research Service (ARS), Center for Grain and Animal Health Research, Stored Product Insect and Engineering Research Unit (SPIERU), 1515 College Ave., Manhattan, KS, USA; ^3^United States Department of Agriculture (USDA), Agricultural Research Service (ARS), Agroecosystem Management Research Unit (AMRU), 251 Filley Hall, UNL East Campus, Lincoln, NE, USA

## Abstract

In agricultural systems, interactions between plants and microorganisms are important to maintaining production and profitability. In this study, bacterial communities in floodwaters of rice fields were monitored during the vegetative and reproductive stages of rice plant development using 16S amplicon sequencing. The study was conducted in the south of Brazil, during the crop years 2011/12 and 2012/13. Comparative analyses showed strong differences between the communities of floodwaters associated with the two developmental stages. During the vegetative stage, 1551 operational taxonomic units (OTUs) were detected, while less than half that number (603) were identified in the reproductive stage. The higher bacterial richness observed in floodwater collected during the vegetative stage may have been favored by the higher concentration of nutrients, such as potassium, due to rhizodeposition and fertilizer application. Eighteen bacterial phyla were identified in both samples. Both communities were dominated by Gammaproteobacteria. In the vegetative stage, Alphaproteobacteria and Betaproteobacteria were more abundant and, in contrast, Bacilli and Clostridia were the more dominant classes in the reproductive stage. The major bacterial taxa identified have been previously identified as important colonizers of rice fields. The richness and composition of bacterial communities over cultivation time may contribute to the sustainability of the crop.

## 1. Introduction

Rice (*Oryza sativa* L.) is the most important cereal crop in the world, feeding more than 50% of the human population. In South America, Brazil is the main producer [1], with flooded rice fields accounting for 50% of the total crop area under cultivation in the country [2]. In flooded rice, the need to maintain adequate water depth throughout most of the crop year characterizes the agricultural system as aquatic in nature. In comparison to other aquatic environments, such as lakes, ponds, and swamps, the environmental conditions in flooded rice fields are relatively unstable due to physical and chemical and biological characteristics that vary according to current agricultural practices and water supplies [3]. The varying physical and chemical properties in this environment could support the growth of microorganisms possessing wide ranges of metabolic plasticity, allowing them to quickly adapt to changing environmental conditions [4]. Thus, the rice ecosystem may be a prime habitat for microorganisms adapted to fluctuating nutritional levels and oxygen and light availability.

The phyla Acidobacteria, Actinobacteria, Bacteroidetes, Chloroflexi, and Proteobacteria have been previously found in soil samples of rice-alfalfa [5] and rice-wheat cropping [6]. In these agroecosystems, rice exudates and nutrients from straw incorporation were shown to influence the bacterial community's composition. Breidenbach and Conrad [7] found a uniform bacterial composition in soil over the rice growing season, with Proteobacteria being the most highly abundant phylum, while Firmicutes represented the fifth most abundant phylum. Firmicutes were also present in relatively low abundance upon the introduction of a maize rotation into an irrigated rice field [8]. Although these microorganisms are common inhabitants of agriculture soils, the low counts of Firmicutes are intriguing considering that this group contains the classes Bacilli and Clostridia, which are often highly abundant in rice agricultural soils where they decompose plant residues using cellulolytic enzymes [9]. Furthermore, the researchers observed higher bacterial 16S rRNA abundance in the flooding stage than in drainage stage, which was ascribed to the rice straw that remained in the field [10].

In the studies mentioned above, the shifts in microbial community structure were governed by environmental factors found in rice fields. These factors can be related to the input of nutrients from water sources, rice residues, and fertilizers, weather conditions, and crop rotation, highlighting the complex network of elements that govern bacterial structure in this agricultural system. Although microbial ecology studies in soil, rhizosphere, and endophytic environments have been previously conducted in rice systems [11–14], the dynamics of bacterial communities in floodwaters from rice fields have not been extensively studied despite the important roles of the floodwaters and microbial communities in rice plant nutrition [15]. Because microbes can contribute strongly to rice nutrition and production, there is much interest in surveying the microbial communities in this flooded agroecosystem and determining how agricultural practices influence the composition and structure of these communities [16]. The purpose of the present study was to investigate the bacterial community structure and composition in floodwaters associated with vegetative and reproductive stages of rice in order to verify if there is a microbial community related to a particular stage of the rice system.

## 2. Materials and Methods

### 2.1. Study Area and Sampling Protocols

**Figure 1:**
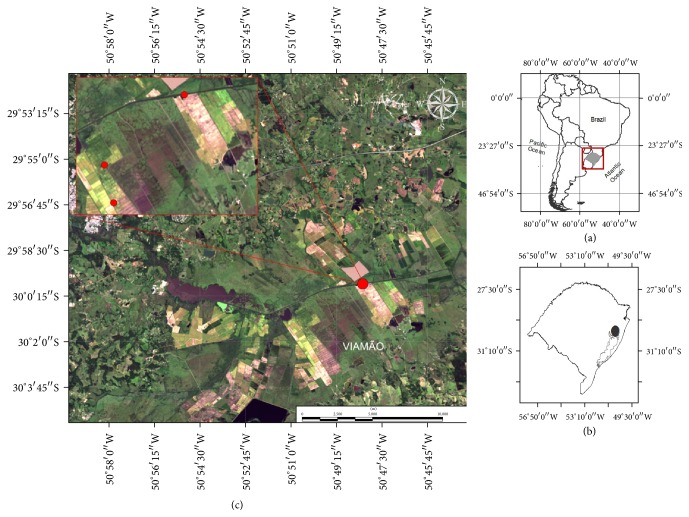
(a) Location of Rio Grande do Sul state, Southern of Brazil. (b) Viamão city, outer coastal plain of Rio Grande do Sul state; (c) study area with the three sampling sites in the flooded rice fields.

Three isolated flooded rice fields were selected for this study. The rice fields were between 164 and 310 ha in area and are located in the city of Viamão (30°04′51′′S, 51°01′22′′W), outer coastal plain, Rio Grande do Sul (RS), Brazil, Figure 1.

Twelve water samples were collected from the three locations above in the agricultural years 2011/12 and 2012/13, six in the vegetative and six in the reproductive stages. One sample from each location and developmental stage was collected during the 2011/12 growing season and another sample was collected during the 2012/13 growing season. The collections were organized as follows: the vegetative (tillering) (October/November) stage was defined by active tillering and coleoptile and radicle (2-3 mm) formation to the beginning of panicle differentiation, while the reproductive stage was defined from the formation until complete maturation of the panicle (December to March) [17]. Each of the 12 individual water samples collected represented a combination of 16 subsamples of 50 mL collected from the top surface of the floodwater to 10 cm in depth [18], thereby maximizing the search for the richness and abundance of the local microbiota. Afterwards, the 16 subsamples from each stage within a location were combined into a single composite water sample of 800 mL for DNA isolation. The 12 samples representing two life stages from three different locations were centrifuged at 12,000 ×g for 15 min at 4°C and the pellets were stored at −20°C for subsequent DNA extraction and analysis.

### 2.2. DNA Extraction and Pyrosequencing

Total DNA was extracted from each of the 12 pellets collected from the floodwater samples from two life stages at three locations using the MoBio Power Soil® DNA Isolation Kit (MoBio, Laboratories Inc., Carlsbad, CA, USA) following the manufacturer's instructions. The DNA yield was measured using a NanoDrop® ND-1000 spectrophotometer (NanoDrop Technologies, Inc. Wilmington, DE, USA) and the DNA quality was verified on a 1.5% agarose gel stained with ethidium bromide (0.1 mg mL^−1^).

Purified DNA from the six replicates collected during the vegetative stage and the six replicates collected during the reproductive stage were pooled together to create a single DNA pool for the vegetative samples and a single DNA pool for the reproductive samples. These two DNA pools (one DNA pool from vegetative samples and one DNA pool from reproductive samples) were used as templates for amplicon pyrosequencing analysis, which was performed by MR DNA Laboratory (Shallow Water, TX, USA), with the goal of investigating the bacterial community structure and composition. The V1–V3 variable regions of the 16S rRNA gene were amplified using primers ill27Fmod (5′-AGRGTTTGATCMTGGCTCAG-3′) and ill519Rmod (5′-GTNTTACNGCGGCKGCTG-3′) [19]. The PCR reactions were prepared with 1 *μ*L of DNA (5 ng *μ*L^−1^) using HotStarTaq Plus Master Mix (Qiagen, Valencia, CA, USA). DNA was initially denatured at 94°C for 3 min followed by 28 cycles of 94°C for 30 s; 53°C for 40 s and 72°C for 1 min; and a final extension at 72°C for 5 min. PCR products of approximately 300 bp were purified using Agencourt Ampure beads (Agencourt Bioscience Corporation, MA, USA). The amplicons were sequenced using 454 GS FLX Titanium chemistry (Roche) following the manufacturer's guidelines. The raw 454 reads generated during the current study have been deposited in NCBI's Sequence Read Archive (SRA) under the accession SRP077313 and are associated with BioProject PRJNA326968.

### 2.3. Protocols and Equipment Used for the Physicochemical Measurements of Water

Water parameters such as temperature, turbidity, and pH were measured from all 12 samples using the Intelligent Meter equipment (INSTRUTHERM PH-1300). In addition, nutrient levels, including total phosphorus (P) and potassium (K), were measured from each of the six water samples collected from the two developmental stages using standard methods [18]. The available P and K were determined using Mehlich-1 solution by molecular colorimetric and flame emission methods, respectively [20, 21].

### 2.4. Data Analysis

The 454 amplicon data from bacterial communities sampled from floodwaters from the reproductive (*n* = 1) and vegetative (*n* = 1) stages were analyzed using the mothur pipeline (version 1.32.1) [22]. In brief, flowgrams were demultiplexed using the trim.flows command and denoised using the shhh.flows command, which is the mothur implementation of PyroNoise [23]. In addition, adapters, barcodes, primers, short reads, and reads containing more than eight homopolymer bases were removed from the dataset with trim.seqs command. Cleaned sequences were aligned to the Silva reference database [24] using the Needleman aligner with the align.seqs command. Chimeric sequences were flagged and removed from the dataset using the uchime algorithm with the reference=self-flag [25]. Then, the remaining high quality sequences were taxonomically classified using the Wang method of the classify.seqs command and the Silva reference taxonomy files. An 80% confidence score was required for all taxonomic assignments. Amplicons classified as mitochondrial, chloroplast, Archaeal, eukaryotic, or unknown in origin were removed from the dataset.

The remaining bacterial sequences were categorized into operational taxonomic units (OTUs) at 3% sequence dissimilarity using the average neighbor algorithm. The consensus taxonomy for each OTU was determined using the classify.otu command. Rarefaction curves were calculated using the rarefaction.single command at a genetic distance of 0.03, while the summary.single command was used to compute various community metrics including the Shannon-Wiener (H′) and Simpson indices, the Chao 1 richness estimator [26], which estimates species richness based on the number of rare OTUs detected in each community, and the Abundance Coverage-based Estimator (ACE), which estimates the number of undetected OTUs based on the abundance and distribution of rare OTUs. Prior to computing rarefaction curves and community diversity/richness metrics, the same number of reads (7,348) were subsampled without replacement from each of the libraries for normalization and to prevent differences in library yields from driving differences between the two communities. The mothur command Libshuff was used to determine whether the bacterial communities from the vegetative and reproductive stages have the same structure with iters set to 1000.

Heatmaps and cluster dendrograms based on Euclidean dissimilarity metrics were built according to the relative abundances of the most common genera in the two communities using the “vegan,” “heatmap.2,” and “cluster” packages [27] in the R statistical computing environment (version 3.1.0). To select the most abundant genera, reads counts for all OTUs assigned to the same genus from both samples were summed together, normalized by relative abundance, and ranked in ascending order. The relative abundances of these genera in floodwaters associated with each developmental stage were log_2_ transformed, *z*-scale standardized, and used for clustering and heatmap analysis. Venn diagrams depicting OTUs found in floodwaters from both developmental stages and and OTUs unique to each developmental stage were constructed using the venn command in mothur.

The physical and chemical characteristics of the floodwater from the rice fields sampled during two agricultural years were analyzed by principal components analysis (PCA) using the following variables: temperature (°C), turbidity (NTU), pH, total phosphorus (P) (mg L^−1^), and potassium (K) (mg L^−1^). Prior to analysis, all variables were log_10_ transformed in order to facilitate comparisons among variables expressed in different units. The correlation matrix with the varimax rotation was set and only autovalues greater than 1 were utilized as criteria for extraction of the principal components. The analysis was performed in the R statistical computing environment (version 3.1.0) using the princomp and biplot functions. The differences among physicochemical parameters between the rice stages were tested using Student's *t*-test in SYSTAT 12 (Systat Software, Inc., CA, USA). *p* values of <0.05 were considered statistically significant.

## 3. Results

### 3.1. Physical and Chemical Characteristics of Rice Field Water

**Figure 2:**
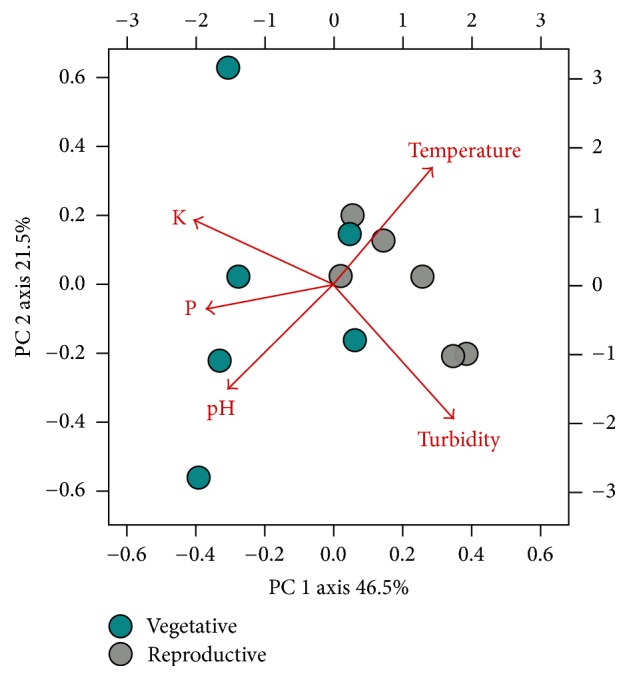
PCA biplot of physical and chemical parameters sampled from rice floodwaters from vegetative and reproductive stages.

Principal components analysis was used to monitor the physical and chemical variations of floodwater of rice fields in vegetative and reproductive stage of two consecutive agricultural years. The physical and chemical patterns of floodwater retrieved from the vegetative and reproductive rice stages are shown in Figure 2.

The first two principal components explained a combined total of 68% of the total variance. Most water samples collected from the vegetative stage were separated from the reproductive stage samples along the PC1 axis. No major differences between locations or agricultural years were noted. In general, vegetative stage samples tended to have higher levels of pH, K, and P compared to the reproductive samples. In contrast, the reproductive samples tended to be more turbid and had higher temperatures compared to the vegetative samples. In addition, some variability was apparent within the water samples collected from each developmental stage, which is likely reflective of the dynamic nature of the physical and chemical variables in rice floodwaters (Figure 2). Temperature and K differed significantly between the two rice development stages. Specifically, the temperature was on average three degrees warmer in floodwaters associated with the reproductive stage. K was almost four times higher in the vegetative compared to the reproductive stage (Table 1).

### 3.2. Composition of the Bacterial Community in Floodwaters over Two Stages of Rice Growth

The pyrosequencing-based analysis generated 25,981 amplicons from the vegetative stage samples and 18,849 amplicons from the reproductive stage samples. After quality filtering and dereplication of identical sequences, 8,483 reads were retained from the water samples collected during vegetative stage and 7,348 reads were retained from samples collected during the reproductive stage. For normalization purposes, 7,348 reads were randomly subsampled without replacement from the vegetative library prior to OTU analysis and taxonomic classification.

**Figure 3:**
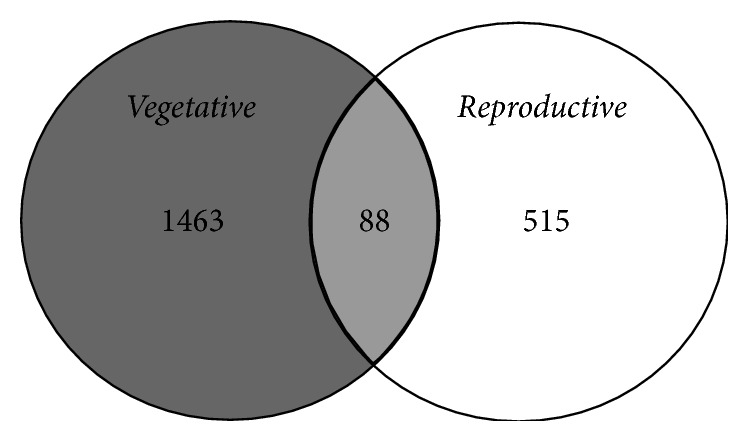
Venn diagram of 16S OTUs shared and unique to floodwater samples collected from vegetative and reproductive stages of rice plants. Sequences were classified into OTUs at 97% similarity using the average neighbor algorithm in mothur.

The structures of the bacterial community differed significantly between the vegetative and reproductive stages of flooded rice (*p* = 0,0001) using Libshuff. In the vegetative stage, 1,551 OTUs were identified, while less than half the number of OTUs (603) were detected in water samples collected from the reproductive stage (Table 2). Further, only 88 (4.08%) of the OTUs detected in this analysis were common to both crop stages, possibly reflecting the dynamic structure of the bacterial communities associated with this environment (Figure 3).

The sequences were distributed among 18 different bacterial phyla. The most species-rich phyla in both the reproductive and vegetative stages were Proteobacteria, which contained 53.34% of the OTUs detected in both samples, Bacteroidetes (16.80% of the OTUs), Actinobacteria (7.84%), and Firmicutes (5.52%). In total, 83.50% of the total OTUs detected in these two communities was associated with these four phyla. Several phyla with lower richness estimates were also detected in these samples, defined as phyla containing less than 1% of the total OTUs detected in this analysis. Based on this criterion, a total of twelve rare phyla were detected including Chloroflexi, Deferribacteres, Deinococcus-Thermus, Fusobacterium, Gemmatimonadetes, OP10, Planctomycetes, SR1, Spirochaetes, TM7, Tenericutes, and Verrucomicrobia, which were detected in both stages of crop development. The phyla Acidobacteria (1.94% of the OTUs) and Cyanobacteria (1.25%) were also present, but at low frequencies (≤2% of the OTUs).

**Figure 4:**
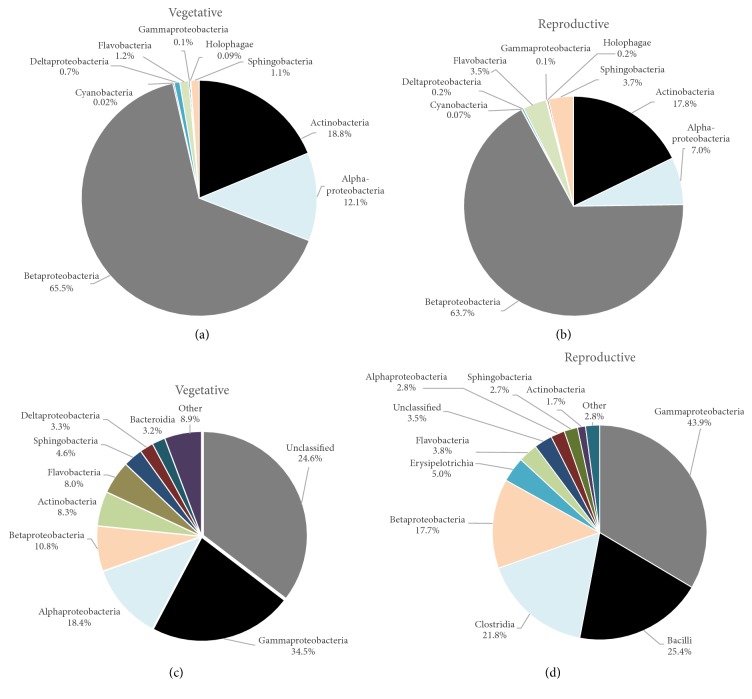
Relative abundances of class level assignments for the 88 shared OTUs in the vegetative stage (a) and reproductive stage (b) and relative abundances of the class level assignments of the 1463 OTUs unique to the vegetative stage (c) and the 515 OTUs unique to the reproductive stage (d). OTUs were taxonomically classified by the Wang method in mothur using an 80% confidence threshold for class level assignments.

In total, 27 different bacterial classes were identified in both developmental stages. In general, the class level abundances of the 88 OTUs shared between the floodwaters from the vegetative and reproductive stages were very similar and were both dominated by Betaproteobacteria, Actinobacteria, and Alphaproteobacteria (Figures 4(a) and 4(b)). In contrast, the class level distributions of OTUs unique to floodwaters sampled from either the vegetative or reproductive stages were drastically different. For example, the community of OTUs unique to the floodwaters of vegetative stage plants was dominated by Gammaproteobacteria (34.5%), Alphaproteobacteria (18.4%), and Betaproteobacteria (10.8%) (Figure 4(c)). Of significance, 24.6% of the amplicon reads derived from OTUs exclusively associated with the vegetative stage could not be conclusively assigned to the class level (Figure 4(c)). In contrast, the community of OTUs unique to floodwaters of reproductive stage plants was dominated by Gammaproteobacteria (43.9% of the amplicon reads), Bacilli (25.4%), Clostridia (21.8%), and Betaproteobacteria (17.7%) (Figure 4(d)).

**Figure 5:**
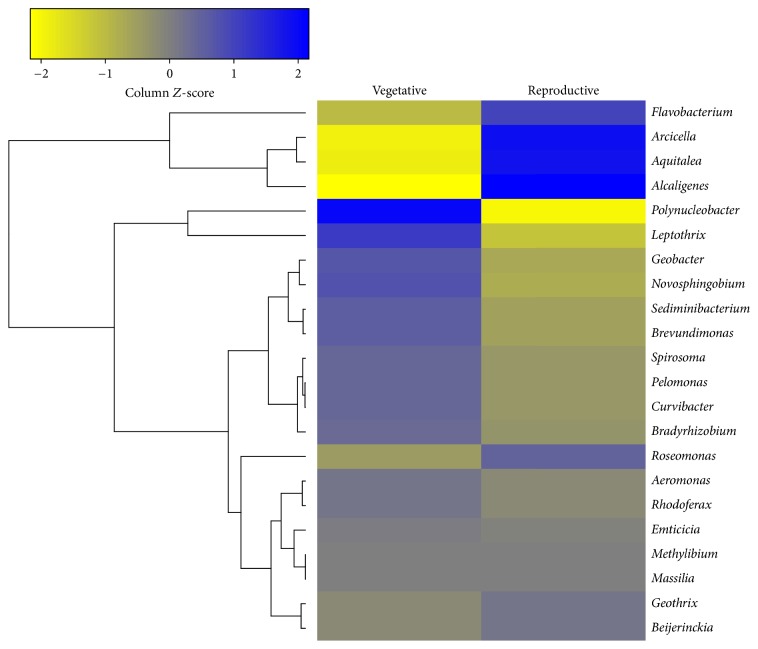
Heatmap and correlation analysis of the 22 bacterial genera found in both the vegetative and reproductive stages of flooded rice. The relative abundances of these genera in floodwaters associated with each developmental stage were log_2_ transformed, scaled by *z*-score, and used for clustering and heatmap analysis. Color intensity is correlated with the relative abundance of each genus in floodwaters associated with the two developmental stages, with blue indicating higher relative abundance and yellow indicating lower relative abundance.

Of the 169 genera identified in the two developmental rice stages, 22 (13%) occurred in both stages (Figure 5).* Beijerinckia*,* Curvibacter*,* Pelomonas*, and* Rhodoferax* occurred in high counts in both phenological stages. In general, the abundances of* Beijerinckia*,* Pelomonas*, and* Rhodoferax* were similar in flood waters from both communities; however, the relative abundance of* Curvibacter* was slightly higher in vegetative stage. The abundances of the genera* Aeromonas*,* Bradyrhizobium*,* Emticicia*,* Massilia*,* Methylibium*, and* Spirosoma* were lower and did not differ significantly between vegetative and reproductive stages (Figure 5).

Figure 5 shows a comparative analysis of the twenty-two most abundant bacterial genera detected in the reproductive and vegetative stages of flooded rice.

**Figure 6:**
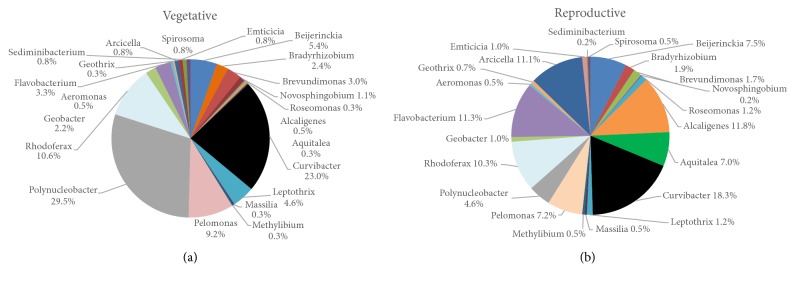
Relative abundances of genus level assignments for the OTUs shared between vegetative stage (a) and reproductive stage (b).

*Polynucleobacter* (29.5%),* Curvibacter* (23%), and* Rhodoferax* (10.3%) represented the most abundant genera in the vegetative stage, while* Curvibacter* (18.3%),* Alcaligenes* (11.8%), and* Flavobacterium* (11.3%) were more abundant in the reproductive stage (Figure 6).

The Shannon-Wiener (H′) index was approximately 25% higher in the water sample collected from the vegetative stage compared to the reproductive stage, suggesting that the vegetative community is richer. In addition, the Simpson index, which represents the probability that two reads selected at random belong to the same OTU, was higher in the sample collected from the reproductive stage. This finding is probably due in part to the lower richness associated with the reproductive stage, the higher number of rare OTUs found in the vegetative stage, and the overabundance of three genera,* Alcaligenes, Curvibacter*, and* Flavobacterium* in the reproductive stage. The Chao 1 richness estimator predicted a total of 2,282 OTUs in the vegetative bacterial community (32% more OTUs than what were detected), while 822 OTUs were predicted in the reproductive bacterial community (27% more OTUs than what were detected). Considering these results, it is clear that a larger number of rare species were associated with water collected from the vegetative stage compared to the reproductive stage (Table 2).

**Figure 7:**
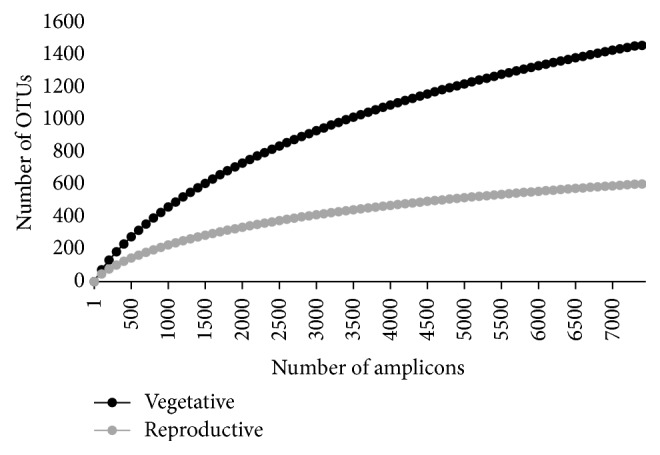
Rarefaction curves generated from 16S amplicons sampled from floodwaters from vegetative and reproductive rice stages. Rarefaction curves were generated using the rarefaction.single command in mothur with freq=100 and iters=10000.

Reflecting the higher richness and higher numbers of rare OTUs, the rarefaction curve of the vegetative stage did not reach complete saturation considering the number of sequences sampled, indicating that additional OTUs will likely be detected with additional sequencing. On the other hand, rarefaction curve of the reproductive stage showed stabilization, indicating that the sequencing effort was sufficient to capture the majority of the OTUs associated with this community (Figure 7).

## 4. Discussion

In flooded rice, the water source used for cultivation is a key component in the management of the crop. Nutrient levels in these water sources can have significant effects on plant nutrition [28] while varying physical and chemical properties in these water sources can also influence the structure, diversity, and taxonomic composition of microbial communities in the floodwaters, which in turn can also profoundly impact plant nutrition. In the rice field plots used for this study, the primary water source contains a high proportion of wastewater input from both urban and agricultural areas [29]. Therefore, a variety of chemical elements enter the floodplain and the temporal fluctuations in the abundance and types of chemical elements entering the fields can have strong influences on the microbial community in rice floodwaters [30].

In the current study, the water samples retrieved from vegetative and reproductive stages showed variations in physical and chemical parameters. The abiotic parameters most strongly associated with the vegetative stage were pH, P, and K and in the reproductive stage, the parameters were temperature and turbidity (Table 1). Of these parameters, temperature (higher in floodwaters from the reproductive stage) and K (higher in floodwaters from the vegetative stage) differed significantly between the two rice development stages. These results reflect differences in microenvironments between the rice plant-phenological stages that may have affected microbial growth and persistence. Supporting the possible impact of these environmental conditions on bacterial communities, significant differences in community composition were observed between the vegetative and reproductive stages of flooded rice, with the diversity and richness values associated with the vegetative stage exceeding those observed in the reproductive stage. Previous research on rice crops reported that the richness and diversity of bacterial communities can change throughout the rice cultivation period in aquatic environments [31–33] and in the soil and rhizosphere environments [34–36]. Although previous studies did not elucidate the environmental factors responsible for these changes in community structure, floodwater characteristics, such as pH, temperature, changes in nutrient profiles, and seasonal variations in fertilizer application, have been related to shifts in microbial community structure and composition over crop development [32]. Additional factors related to plant growth can also cause shifts in microbial communities. For example, Okabe and coworkers [31] determined that plant growth varies with the incidence of sun light, which, in turn, can impact the water temperature and algal growth. Higher photosynthesis rates associated with algal proliferation and also plant growth [34] can ultimately lead to increased levels of photosynthates in the water, altering the water pH and potentially altering the bacterial community.

A total of 2,066 OTUs at 3% dissimilarity were identified in floodwaters collected from the reproductive and vegetative developmental stages of rice. In flooded rice systems, the short spacing between the plants, the low depth of the floodwaters (10 cm deep), and soil management regimes can increase the levels of suspended soil particles in the floodwaters [37], which is in contrast to other more static aquatic ecosystems. Due to the high turbidity and the presence of soil in the floodwaters, it is expected that bacterial groups associated with soil, such as Proteobacteria, Bacteroidetes, Actinobacteria, and Firmicutes, will be present in the floodwaters of rice fields, contributing to the total bacterial diversity [38]. Supporting this hypothesis, the major phyla identified in the floodwaters associated with both developmental stages were Proteobacteria, Bacteroidetes, Actinobacteria, and Firmicutes, while Acidobacteria and Cyanobacteria were less frequent. The same phyla were highly abundant in other rice habitats [7], and specifically in water samples collected from flooded rice crops [3, 32, 39]. Furthermore, Proteobacteria (alpha- and beta-subdivision), Bacteroidetes, and Actinobacteria were also cited as the major groups of bacteria found in freshwater environments [39]. In unstable environments, such as rice fields, Proteobacteria may contribute to the growth, development, and physiology of rice plants due to the presence of plant growth-promoting bacteria, which are often able to induce callus in rice and produce phytohormones [40]. According to Mhuantong et al. [16], the presence of these bacterial groups in a variety of freshwater ecosystems is related to their metabolic plasticity to decompose an assortment of organic matter.

Despite the presence of several common phyla in floodwaters sampled from both developmental stages, only 88 (4.08%) of the OTUs were common between vegetative and reproductive stages, suggesting that the dynamics of the bacterial communities in floodwaters change throughout the growing season. These changes could reflect temporal variations in carbon availability. For example, in a previous study, Aulakh and coworkers [41] documented higher exudation rates over the course of rice plant development, in which sugars in the exudates were eventually replaced by organic acids in later developmental stages. Both sugars and organic acids are essential for bacterial nutrition and contribute to the process of microbial colonization [42]. Furthermore, when soluble carbon is released into the rhizosphere, microbes in the soil quickly mineralize the available carbon [43]. Thereby, the amount of root exudates in the soil could affect the relative amounts of carbon and nitrogen available for microbial growth [44], promoting transitions in the size of populations and structure of microbial communities in the rhizosphere [45]. Nevertheless, the composition and quantity of exudates are dynamic in time and space, and therefore it becomes difficult to resolve the role of a single component in the structure of microbial community [46].

The richest bacterial classes found in the vegetative stage were Gammaproteobacteria (276 OTUs), Alphaproteobacteria (237 OTUs), and Betaproteobacteria (211 OTUs), while richness was the highest in Gammaproteobacteria (24 OTUs), followed by Bacilli (46 OTUs) and Clostridia (18 OTUs) during the reproductive stage. In flooded plains, the adherence of Betaproteobacteria representatives to particles can play an important role in their transport from the terrestrial environment to floodwater [47], while the rice phyllosphere is greatly colonized by Alphaproteobacteria class [48]. Thus, Beta- and Alphaproteobacteria represent relevant bacteria groups in the soil whose abundances are strongly linked to carbon supply [49]. In flooded rice systems, these bacterial groups decompose organic matter derived from rice straw and other fermentable crop residues along with fertilizer where organic molecules (humic substances) can act as an electron donor during microbial respiration [47].

Gammaproteobacteria were present in high richness and abundance in floodwaters associated with both rice developmental stages. Overall, members from the phylum Proteobacteria are often the most abundant freshwater prokaryotes, but Gammaproteobacteria are usually present in low or transient abundances [38], although they were previously found in high amounts in the rice phyllosphere [40]. This finding suggests that specific environmental factors associated with the rice agroecosystem, such as the source of water used to irrigate the fields and the turbidity of the floodwaters, provided conditions for the survival and persistence of members of this phylum with the entrance of nutrients. This class hosts the genera* Escherichia*,* Salmonella*,* Yersinia*,* Vibrio*, and* Pseudomonas* characterized by their metabolic plasticity that can be adapted to varying levels of temperature, oxygen supply, and nutritional requirements. Hence, members of the class Gammaproteobacteria often dominate microbial assemblages after nutrient enrichment [50]. In addition, the members of this group contain pathways for degradation of carbohydrates, amino acids, and xenobiotics that may provide competitive advantages under certain ecological condition [51]. Furthermore, the interaction between Gammaproteobacteria members and rice plants may be beneficial to both parties, with the leaf exudates providing nutrients that support microbial growth and microorganisms assisting better rice yield [40].

*Beijerinckia*,* Curvibacter*,* Pelomonas*, and* Rhodoferax* were the four most abundant genera in both stages of the crop. These bacterial groups are often associated with high availabilities of sugar (e.g., glucose) [52]. In agricultural and freshwater environments,* Beijerinckia* are reported as growth promoters in plants [53], while the genomes of* Curvibacter* spp. often code for large numbers of sugar transporters [54], enhancing its ability to explore nutrients from the environment. Other genera, such as* Pelomonas* sp. and* Rhodoferax* sp., were found in approximately the same proportion in both rice stages. Previous rDNA 16S amplicon analysis showed that* Rhodoferax* spp. are common in diverse freshwater systems [55]. Some species of the genera* Rhodoferax* are nitrate-reducers [56], while* Pelomonas* spp. often code for machinery required for nitrogen fixation (*nifH* gene) [57]. Thus, the OTUs assigned to these genera in the current study may contribute to nitrogen flux in flooded rice fields. In contrast, the abundance of the genus* Polynucleobacter* differed significantly in floodwaters from the two life stages. Its abundance was significantly higher in the vegetative stage (29.5%) compared to reproductive stage (4.6%). This result could suggest that the OTUs derived from this genus are not well adapted to environmental conditions associated with floodwaters collected from reproductive stage plants.* Polynucleobacter* members often dominate planktonic freshwater communities [58], where pH, conductivity, and dissolved organic carbon concentration play an important role in colonization [59]. Also, flagellate grazing and the consumption of low molecular weight substrates are related to the survival of* Polynucleobacter* in freshwater environments [60, 61].

Rises in temperature can increase the abundance of prokaryotes of the natural communities by enhancing their growth rates [62]. The abundance of the phylum Firmicutes (5.52%) found in the reproductive stage is consistent with the abundance of this phylum commonly found in the rice fields [63]. However, the presence of these spore-forming populations in the reproductive stage may be related to the characteristics of the organic matter available associated with this developmental stage [63] which may vary from straw residues and root residues [64]. Species belonging to the class Clostridia are able to express enzymes that degrade the cellulose and sugar transport systems to quickly uptake sugars released from plant cell walls [65]. In rice field soil, these organisms were responsible for the decomposition of straw residue at 15 and 30°C [64]. From this basis, conditions found in reproductive stage such as the high temperature and carbon available to be degraded may have promoted the proliferation of OTUs belonging to Gammaproteobacteria and Firmicutes.

Rhizodeposition occurs continuously during the lifetime of plants in the form of water soluble exudates, secretions, and plant dead cells. This process contributes as a primary source of energy and nutrients for microbes in the floodwaters [66]. In future studies, the release of carbon compounds from rice roots should be considered in the investigation of the factors responsible for shaping the structure of the bacterial communities in floodwaters of rice systems [34]. With respect to environmental and nutritional conditions during the vegetative stage, approximately 20% of the absorbed photosynthetic carbon is released through rhizodeposition [67]. Moreover, during the reproductive stage, the plants release lower amounts of carbon compounds in their exudates because they devote the majority of their energy to seed production [68, 69]. Breidenbach and Conrad [7] investigated the bacterial community of the soil from rice fields using 16S rDNA amplicon analysis and also profiled the expression level of various taxa throughout the vegetative, reproductive, and maturation stages. According to quantitative PCR, the authors detected elevated bacterial abundances in soil from the reproductive stage, but there were no major differences between the bacterial community compositions during the two growth stages. Changes in bacterial abundance between soil collected from the vegetative and reproductive stages were strongly attributed to decreased levels of root exudates by plants nearing maturity. In general, the abundance of bacteria in the soil was dynamic throughout the development of rice plants; however, no microbial taxa appeared to be exclusively associated with a particular stage of development.

Heterotrophic microorganisms, which represent a large portion of the rice field microbiome [70, 71], are dependent on carbon substrates for their energy supply. The vegetative stage (tillering) is the most vigorous growth period of the rice plant [72]. As plant biomass and growth increase, intensifications of organic carbon secretions can also occur [73]. Thus, considering that the higher carbon levels could support the persistence of a larger diversity of microbes, the higher levels of exudates could contribute to the higher richness observed in this study in floodwaters collected from vegetative stage. Moreover, the use of urea in fertilizers, which is applied mainly during the vegetative stage, can affect the concentrations of available nutrients, especially NH_4_^+^, which is the main form of available N in the soil [74]. These factors, alone or in combination, may have helped to shape the bacterial communities, increasing its diversity in the vegetative stage of crop. In that sense, the release of root exudates can influence the dynamics of bacterial communities during crop development and should be investigated as a contributor to bacterial community dynamics in rice floodwaters.

## 5. Conclusion

This study revealed that the communities associated with rice floodwaters were variable and differed between floodwaters from the vegetative and reproductive stages of rice. The higher bacterial richness observed in floodwaters associated with vegetative plants may have been favored by the higher concentration of nutrients (for example, phosphorus and potassium) due to changes in rhizodeposition and crop management. OTUs from the phylum Proteobacteria were present in floodwaters collected from both developmental stages, indicating the persistence of this phylum in flooded rice ecosystems. Moreover, the predominance of the class Gammaproteobacteria and the occurrence of the phylum Firmicutes in the reproductive stage demonstrated that the environment was favorable to microbes that can persist longer in paddy fields where they are able to utilize the degradable fraction of organic materials and survive in waterlogged and dry conditions. However, further investigations regarding the composition and quantification of root exudates and the impacts of rice exudates on microbial communities in rice fields are essential to decipher the linkage between the microbial community dynamics and rice plant over cultivation time.

## Figures and Tables

**Table 1:** Mean values and corresponding standard deviations, for the physicochemical parameters measured in the water samples in relation to the rice stages.

Rice stages		Temperature^*∗*^ (°C)	pH	Turbidity (NTU)	Total P (mg/L)	Total K (mg/L)^*∗*^
Vegetative		30.20	6.50	4.30	0.65	14.70
		24.06	6.21	34.16	0.44	31.75
		22.73	7.43	68.83	0.84	12.34
		29.40	6.70	39.86	0.01	11.77
		23.60	6.50	37.56	0.02	4.29
		27.20	7.43	42.20	1.30	11.59
	Mean ± SD	26.20 ± 3.18	6.80 ± 0.51	37.82 ± 20.60	0.54 ± 0.49	14.41 ± 9.19

Reproductive		28.70	6.17	108.00	0.05	4.50
		31.30	6.70	41.80	0.05	4.20
		27.30	6.20	253.20	0.01	1.89
		29.20	6.43	93.10	NA	6.10
		29.60	6.50	27.55	0.17	5.04
		30.80	6.50	391.00	0.14	1.62
	Mean ± SD	29.48 ± 1.44	6.42 ± 0.20	152.44 ± 141.70	0.08 ± 0.06	3.89 ± 1.77

NA: not available; SD: standard deviation; *∗* means differed significantly (*p* < 0.05).

**Table 2:** Richness estimators (Chao 1 and Ace) and diversity indices of Shannon-Wiener (H′) and Simpson from bacterial communities sampled from floodwaters during reproductive and vegetative stages of rice using 16S amplicons. A 3% nucleotide dissimilarity was used for OTU discrimination.

Crop stage	Number of sequences			3% divergence				
	Raw sequences	Analysed sequences	Normalization	OTUs	Chao 1	Ace	Shannon-Wiener (H′)	Simpson
Vegetative	25,981	8,483	7,348	1,551	2,282	2,266	5.77	0.03
Reproductive	18,849	7,348	7,348	603	822	842	4.28	0.05
